# Risk factors for the occurrence and protraction of patellar and patellar tendon pain in children and adolescents: a prospective cohort study of 3 years

**DOI:** 10.1186/s12891-022-05349-y

**Published:** 2022-04-26

**Authors:** Manato Horii, Ryuichiro Akagi, Sho Takahashi, Shotaro Watanabe, Yuya Ogawa, Seiji Kimura, Satoshi Yamaguchi, Seiji Ohtori, Takahisa Sasho

**Affiliations:** 1 Department of Orthopedic Surgery, Graduate School of Medicine, Chiba University, 1-8-1 Inohana, Chuo-ku, Chiba, Chiba 260-8670 Japan; 2 Sports Medics Center, Chiba University Hospital, 1-8-1 Inohana, Chuo-ku, Chiba, Chiba 260-8670 Japan; 3 Clinical Research Support Center, The Jikei University School of Medicine, 3-25-8 Nishi-Shimbashi, Minato-ku, Tokyo, 105-8461 Japan; 4 Graduate School of Global and Transdisciplinary Studies, Chiba University, 1-33 Yayoi, Inage-ku, Chiba, Chiba 263-8522 Japan; 5 Center for Preventive Medical Sciences, Musculoskeletal Disease and Pain, Chiba University, 1-8-1 Inohana, Chuo-ku, Chiba, Chiba 260-8670 Japan

**Keywords:** Patellar and patellar tendon pain, Children, Adolescents, Pediatrics, Physical activity, HSS Pedi-FABS

## Abstract

**Background:**

Patellar and patellar tendon pain is a common limitation to children’s participation in social and physical activities. Some factors have been implicated in the occurrence and protraction of knee pain, but the causal relationship is unknown. The purpose of this study was to investigate whether participants’ physical characteristics and activity level are risk factors for the occurrence and protraction of patellar and patellar tendon pain in children and adolescents.

**Methods:**

A three-year prospective cohort study was conducted with healthy students who were aged 8–14 years old, in Japan. Height, weight, heel-buttock distance, straight leg raising angle, and dorsiflexion angle of the ankle joint were collected as individual physical factors at the beginning of each year. The presence of self-reported patellar and patellar tendon pain and the Hospital for Special Surgery Pediatric Functional Activity Brief Scale (HSS Pedi-FABS) was collected every month. Protraction was defined as either (1) pain lasting for more than three continuous months or (2) recurrent pain after more than three months of complete recovery. Participants who did not have any pain at the beginning of the observation period were included in the analysis. We analyzed the odds ratio (OR) of pain occurrence within a year of registration and protraction throughout the study period for all physical factors and HSS Pedi-FABS.

**Results:**

We included 1133 participants in the analysis and 252 participants developed knee pain within a year. 34.8% of participants with pain experienced protraction during the follow-up period. A high HSS Pedi-FABS significantly predicted knee pain occurrence (OR 1.03, 95% confidence interval [CI] 1.01–1.05) and protraction (OR 1.03, 95% CI 1.00–1.05). In addition, younger children and girls were at a significantly higher risk of patellar and patellar tendon pain protraction (age, OR 0.81, 95% CI, 0.73–0.90; sex, OR 1.69, 95% CI, 1.09–2.64). Other physical factors did not significantly predict the occurrence or protraction of knee pain.

**Conclusions:**

This study showed that a greater physical activity level was a risk factor for the occurrence and protraction of patellar and patellar tendon pain in childhood. In addition, younger age and female sex predicted higher risk of protraction of pain.

**Supplementary Information:**

The online version contains supplementary material available at 10.1186/s12891-022-05349-y.

## Background

The knee is one of the most common sites of musculoskeletal pain in children and adolescents [1]. A cross-sectional study reported that 23% of adolescents who were aged 10–17 year had knee pain [2]. Patellar tendon related pain in children and adolescents is a common phenotype and is usually considered benign and self-limiting [3–5]. Rest, medication, and physical therapy are typically successful treatments in more than 90% of patients [6]. However, some children experience severe or chronic pain, leading them to change sports or limit physical activity and social participation [7, 8]. Therefore, prevention of occurrence and protraction of patellar and patellar tendon pain is necessary and it is essential to recognize the characteristics of children and adolescents who are likely to have and develop chronic knee pain.

Studies have reported several factors associated with knee pain in children and adolescents. Excess weight is correlated with increased knee pain and knee joint dysfunction, as well as pain protraction [9, 10]. Another study reported lower extremity muscle tightness as a risk factor for knee pain [11, 12]. In Denmark, adolescents with knee pain had significantly higher levels of participation in leisure sports than those without knee pain [13]. However, since the previous reports were mainly based on retrospective or cross-sectional studies, it remains unclear whether these factors are the cause or the result of persistent knee pain. To date, there are only a few reports based on adult studies that have investigated the causes of chronic knee pain, and clinicians have little empirical evidence to inform clinical decisions to make recommendations to younger patients.

The purpose of this study was to describe the incidence of patellar and patellar tendon pain in Japanese children and adolescents who were aged 8–14 years and identify the risk factors associated with its occurrence and protraction.

## Methods

### Study design

This study was approved by the Institutional Review Board and was conducted as a prospective cohort study. The school year in Japan begins in April and ends in March. Following a pilot study phase from April 2016 to March 2017, the surveillance period was open from April 2017 to February 2020.

**Fig. 1:**
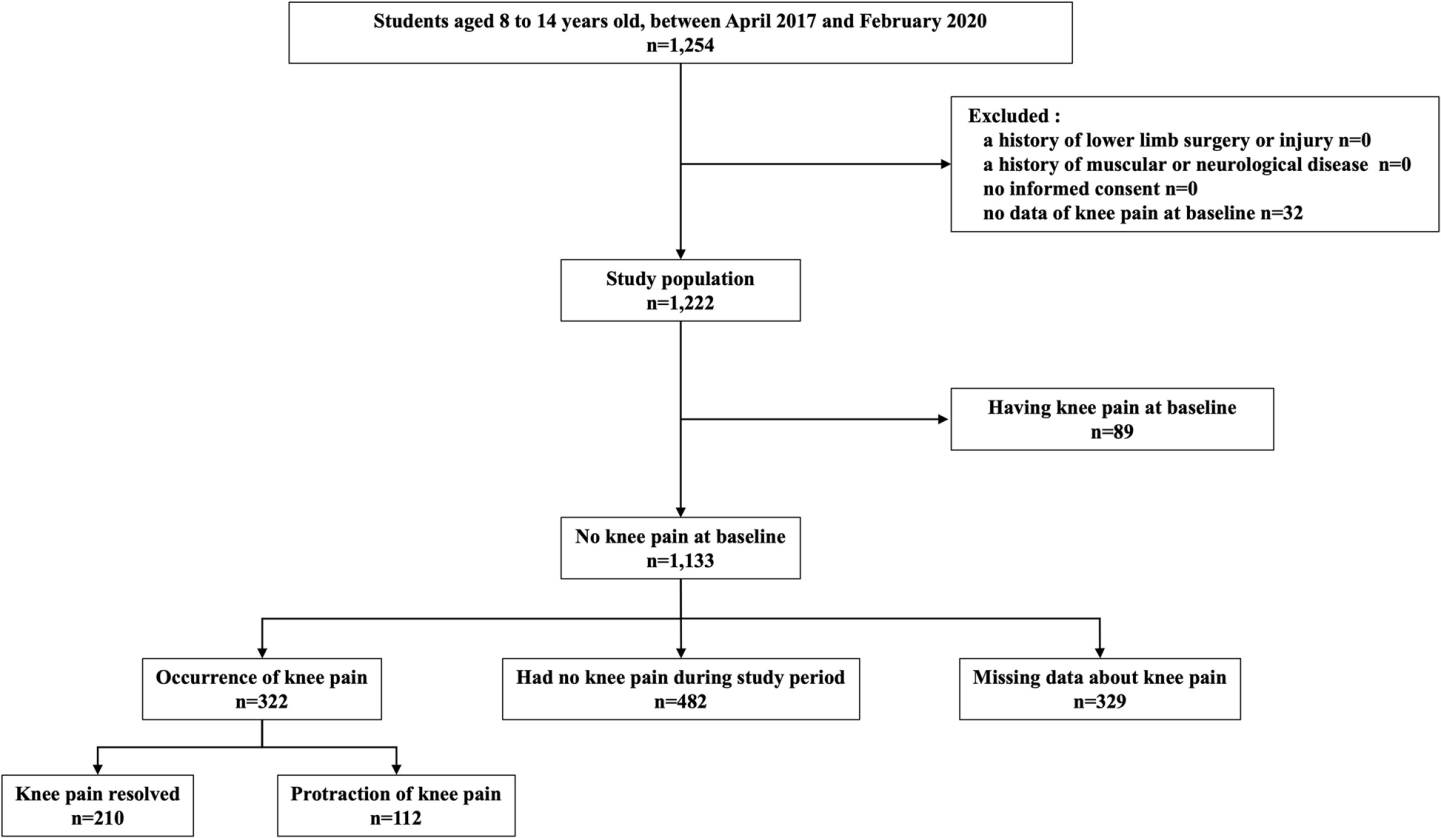
Flow diagram of this study

Students who were in the third to sixth grades of elementary school (8–11 years old) and the first to third grades of junior high school (12–14 years old) were eligible for enrollment in the study. Written informed consent was obtained from all participants and their guardians before participation in the study. None of the students skipped or repeated grades during the study period. Participants with lower limb trauma at the time of baseline examination, musculoskeletal or neurological disorders that made it impossible to perform the physical examination alone or to walk independently, history of lower limb surgery, or those without baseline data regarding the presence of knee pain, were excluded (Fig. 1).

### Data collection

Data were collected at the school under the supervision of teachers, when required. Participant demographic data, such as sex and age, were recorded at the time of initial participation in the study. Height and weight data were recorded at the beginning of each semester in April, September, and January.

In the beginning of each fiscal year in April, from 2017 to 2019, a direct musculoskeletal examination by orthopedic surgeons and physical therapists was conducted with a two-day schedule. The examination aimed to screen children and adolescents for musculoskeletal problems, presence of knee pain, and lower limb tightness. There were approximately 850 participants and 30–34 examiners per year. Each examiner received three to four training sessions prior to the medical assessment for accurate and uniform evaluation. Three examiners formed a group and each group examined approximately 40 participants per day. The examination was conducted by at least two examiners per participant to allow for one examiner to record the measurements while the other performed the examinations.

Knee pain in this study was defined as the presence of tenderness by gentle palpation of the supra- and infra-patellar poles and the tibial tubercle in each knee [14]. These three inspection sites were chosen as they are anatomically easy to define and are common sites of patellar and patellar tendon pain in children [13]. Knee pain was marked as positive if the participant felt pain to any degree by gentle palpation in at least one of these sites.

Three items were measured to assess lower limb tightness, as previously reported: 1) heel-buttock distance (HBD, cm) [11]; 2) straight leg raising angle (SLRA, degrees) [11]; and 3) dorsiflexion angle of the ankle joint at the knee in the extended position (DFA, degrees) [15]. With the participant in the prone position, the examiner measured the HBD by bending the participant’s knees, individually, as far as possible until the examiner felt resistance. The distance from the heel to the buttock was measured using a standard ruler, and the distance was recorded in centimeters to the first decimal point. Thereafter, the participant was placed in a supine position, and each leg was raised with the knees extended to measure the SLRA, individually. The angle of the inspection table with the femoral shaft was measured using a large custom-made protractor and recorded in one-degree increments. Finally, the maximum DFA was measured on each side with the knee extended by setting the stationary arm of the goniometer parallel to the fibular shaft and the movement arm parallel to the fifth metatarsal and was recorded in one-degree increments. In the analysis, we handled the values that we judged to be associated with more tightness, such as higher HBD, and lower SLRA and DFA, as variables.

On the final week of each month, school teachers distributed the questionnaire to their students. The participants were required to report the presence of knee pain by palpating the indicated points by themselves while looking at the knee schematic diagram on the questionnaire. In addition, they answered a questionnaire to collect data on the degree of physical activity. The participants submitted their questionnaires to school teachers within one week. All participants received instructional sessions to identify the self-inspection sites of their knee and an instruction paper with photos of the inspection sites were distributed prior to the study. Physical activity was quantified using the Hospital for Special Surgery Pediatric Functional Activity Brief Scale (HSS Pedi-FABS) [16]. The HSS Pedi-FABS is a patient-reported outcome measure, with eight validated items designed to quantify movement in children who were aged 10–18 years [17]. The scale ranges from 0 to 30, depending on how physically active the respondents were during the past month.

### Occurrence and protraction of knee pain

Participants without knee pain at baseline were assessed for the occurrence and protraction of knee pain. The occurrence of knee pain was defined as the first time the participant indicated positive pain on any of the points of palpation.

Protracted knee pain was defined as; 1) chronic pain that lasted for more than three months or 2) recurrence after the pain had disappeared for more than three months, according to a previous report [18].

### Statistical analysis

Summary statistics for baseline variables were created using frequencies and proportions for categorical data and mean and standard deviation (SD) for continuous variables.

To analyze the occurrence of knee pain within one year after the beginning of observation, we used a multivariable logistic regression analysis to calculate the odds ratio (OR) and investigated the effects of each factor. The model was adjusted for age, sex, body mass index (BMI), HBD, SLRA, DFA, and HSS Pedi-FABS at the baseline. Covariates for adjustment were selected based on clinical significance and previous studies.

In the analysis of chronic knee pain, OR was calculated using the generalized estimating equations for the multivariable logistic regression model, and the effect of each factor was investigated. The model was adjusted for age, sex, BMI, HBD, SLRA, DFA, and HSS Pedi-FABS. Sex was handled as a binary variable divided into boys and girls, and we calculated the OR of girls relative to that of the boys. Covariates for adjustment were selected based on clinical significance and previous studies. Participants with missing data about knee pain during the study period were considered to have dropped out starting from the month the missing data was found. The presence of missing data might have biased the results. Therefore, we performed a sensitivity analysis for both occurrence and protraction of knee pain by complementation referring to the previous month of missing data. Complementation was considered impossible if missing data continued for more than two months in a row.

All p-values were two-sided. Statistical significance was set at p < 0.05. All statistical analyses were performed using SAS software (version 9.4; SAS Institute, Cary, NC, USA).

## Results

We enrolled 1254 students; among them, 32 without knee pain data at baseline were excluded (Fig. 1). Of the 1222 students, 1133 did not have pain at baseline. Table 1 shows the characteristics of the participants sorted by the presence of knee pain at baseline. Of the 1133 students, 252 participants had knee pain within a year, and 329 participants had at least one missing data on the presence of knee pain. The characteristics of the participants who were included in the analysis and those who were excluded due to missing data are summarized in Table 2. There were 322 students who newly developed knee pain during the study period, 55 students developed persistent knee pain, and 57 students developed recurrent knee pain. The median duration of knee pain persistence was 7 months (Interquartile range 3 to 29 months).

**Table 1:** Characteristics of the participants at baseline in this study

	Total (*n* = 1222)	Knee pain (−) (*n* = 1133)	Knee pain (+) (*n* = 89)
Age (y)			
8	305 (25.0)	292 (25.8)	13 (14.6)
9	110 (9.0)	106 (9.4)	4 (4.5)
10	122 (9.9)	109 (9.5)	13 (14.6)
11	110 (9.0)	102 (9.0)	8 (9.0)
12	282 (23.1)	262 (23.1)	20 (22.5)
13	150 (12.3)	134 (11.8)	16 (18.0)
14	143 (11.7)	128 (11.3)	15 (16.9)
Sex			
Girls	613 (50.2)	582 (51.4)	31 (34.8)
Boys	609 (49.8)	551 (48.6)	58 (65.2)
BMI (kg/m^2^)	17.4 ± 2.5	17.4 ± 2.6	17.4 ± 2.5
Lower limb tightness			
Rt HBD (cm)	1.3 ± 2.8	1.3 ± 2.8	1.5 ± 3.2
Lt HBD (cm)	1.3 ± 2.8	1.3 ± 2.7	1.7 ± 3.7
Rt SLRA (deg)	76.6 ± 13.3	76.8 ± 13.1	74.0 ± 14.7
Lt SLRA (deg)	76.6 ± 13.5	76.9 ± 13.3	73.5 ± 15.3
Rt DFA (deg)	15.6 ± 6.9	15.7 ± 6.9	14.0 ± 7.0
Lt DFA (deg)	15.3 ± 6.8	15.3 ± 6.8	14.1 ± 7.1
HSS Pedi-FABS	15.5 ± 8.1	15.5 ± 8.1	15.2 ± 8.7

Data on age and sex are presented as number (prevalence), while BMI, HBD, SLRA, DFA, and HSS Pedi-FABS are presented as mean ± standard deviation

*BMI* Body mass index, *HBD* Heel-buttock distance, *SLRA* Straight leg raising angle, *DFA* Dorsiflexion angle of the ankle joint with knee extension, *HSS Pedi-FABS* The Hospital for Special Surgery Pediatric Functional Activity Brief Scale

**Table 2:** Characteristics of the participants who had missing data about knee pain or not

	Missing data (−) (*n* = 804)	Missing data (+) (*n* = 329)
Age (y)		
8	264 (32.8)	28 (8.5)
9	96 (11.9)	10 (3.0)
10	89 (11.1)	20 (6.1)
11	20 (2.5)	82 (24.9)
12	210 (26.1)	52 (15.8)
13	120 (14.9)	14 (4.3)
14	5 (0.6)	123 (37.4)
Sex		
Girls	407 (50.6)	175 (53.2)
Boys	397 (49.4)	154 (46.8)
BMI (kg/m2)	17.1 ± 2.5	18.3 ± 2.5
Lower limb tightness		
Rt HBD (cm)	1.0 ± 2.3	2.2 ± 3.7
Lt HBD (cm)	0.9 ± 2.2	2.1 ± 3.6
Rt SLRA (deg)	77.4 ± 13.6	75.3 ± 11.6
Lt SLRA (deg)	77.5 ± 13.8	75.4 ± 11.7
Rt DFA (deg)	16.0 ± 7.1	14.8 ± 6.5
Lt DFA (deg)	15.6 ± 7.0	14.6 ± 6.3
HSS Pedi-FABS	15.9 ± 7.9	14.6 ± 8.4

Data on age and sex are presented as number (prevalence), while BMI, HBD, SLRA, DFA, and HSS Pedi-FABS are presented as mean ± standard deviation

*BMI* Body mass index, *HBD* Heel-buttock distance, *SLRA* Straight leg raising angle, *DFA* Dorsiflexion angle of the ankle joint with knee extension, *HSS Pedi-FABS* The Hospital for Special Surgery Pediatric Functional Activity Brief Scale

Table 3 shows the OR and confidence interval (CI) for each factor at the baseline for the occurrence of patellar and patellar tendon pain. High HSS Pedi-FABS were significant risk factors of pain occurrence. Table 4 shows the ORs and CIs of each factor for pain protraction. High HSS Pedi-FABS was also significantly risk factor of pain protraction. In addition, younger children and girls were more associated with pain protraction compared to their counterparts.

**Table 3:** The OR and CI of the knee pain occurrence within a year by each factor

	OR [95% CI]	*P* value
Age	0.92 [0.84, 1.00]	0.06
Sex ^a^	1.03 [0.72, 1.47]	0.87
BMI	1.00 [0.93, 1.08]	0.97
HBD	1.04 [0.97, 1.11]	0.29
SLRA ^b^	0.93 [0.82, 1.06]	0.30
DFA ^b^	0.99 [0.88, 1.11]	0.86
HSS Pedi-FABS	1.03 [1.01, 1.05]	0.01

*BMI* Body mass index, *HBD* Heel-buttock distance, *SLRA* Straight leg raising angle, *DFA* Dorsiflexion angle of the ankle joint with knee extension, *HSS Pedi-FABS* The Hospital for Special Surgery Pediatric Functional Activity Brief Scale; OR, odds ratio; CI, confidence interval

^a^Reference group is boys

^b^Units of SLRA and DFA are in 10° and 5° increments, respectively

**Table 4:** The ORs and CIs of the protraction of knee pain by each factor

	OR [95% CI]	*P* value
Age	0.81 [0.73, 0.90]	<0.01
Sex ^a^	1.69 [1.09, 2.64]	0.02
BMI	1.03 [0.93, 1.14]	0.56
HBD	0.99 [0.90, 1.09]	0.77
SLRA ^b^	0.86 [0.73, 1.00]	0.06
DFA ^b^	1.07 [0.95, 1.20]	0.26
HSS Pedi-FABS	1.03 [1.00, 1.05]	0.02

*BMI* Body mass index, *HBD* Heel-buttock distance, *SLRA* Straight leg raising angle, *DFA* Dorsiflexion angle of the ankle joint with knee extension, *HSS Pedi-FABS* The Hospital for Special Surgery Pediatric Functional Activity Brief Scale; OR, odds ratio; CI, confidence interval

^a^Reference group is boys

^b^Units of SLRA and DFA are in 10° and 5° increments, respectively

The sensitivity analysis results were shown in Additional files 1 and 2, and were similar to the initial analysis.

## Discussion

The most important finding of our study was that children and adolescents who were aged 8–14 years with higher levels of physical activity have a generally higher risk of developing chronic patellar and patellar tendon pain. In addition, the prevalence of chronic knee pain throughout the observation periods was 34.8% in children and adolescents who were aged 8–14 years, and the average period of continuous pain was 7 months. Younger children and girls were at a higher risk of protracted knee pain than older adolescents and boys. Most previous studies examined non-specific knee pain in children and adolescents, or young athletes with patellar and patellar tendon pain, and compared the characteristics and sports activity levels between participants with and without knee pain [12, 19]. The novelty of our study is that we focused on children and adolescents without pain at baseline and conducted a longitudinal study to investigate the risk factors for its occurrence and protraction.

There have been several studies on the risk factors for patellar and patellar tendon pain in children and adolescents. A cross-sectional study compared lower limb tightness in adolescents between those with knee pain and without knee pain and revealed that adolescents with knee pain had high HBD and low DFA [12]. Another study reported the involvement of hamstring tightness in knee pain [7]. Our study showed no obvious association between the occurrence of knee pain and HBD, SLRA, or DFA. The difference between the current study and previous studies may be that the previous study focused only on athletic adolescents because even the participants without knee pain had tighter lower limbs, with a mean HBD of 6 cm and an SLRA of 71 degrees, compared to our study participants (mean HBD 1.0 cm and SLRA 77 degrees). Other studies have shown an association between patellar and patellar tendon pain and physical activity [20, 21]. In a cross-sectional study, Tomaru et al. showed that the longer the exercise time, the higher the proportion of knee pain in elementary and junior high school students [21]. This study was the first to evaluate the relationship between physical activity level and knee pain. High HSS Pedi-FABS was a significant risk for knee pain occurrence in our study. These results suggest that patellar and patellar tendon pain occurrence may be prevented by controlling the amount of physical activities.

The prevalence of chronic knee pain in children and adolescents has been reported to be 31–40% in studies with follow-up periods of 1–5 years [22, 23]. In our study, 34.8% of Japanese children and adolescents suffered from chronic pain, which is in line with the results from other cohorts. Some previous reports have studied the risk factors for chronic knee pain in children and adolescents. Several reports revealed that women tended to have more knee pain than men [7, 24]. In a cross-sectional study including 967 children and adolescents in Finland, the relationship between age, sex, weight, and the frequency of chronic knee pain was investigated [25]. The investigators concluded that adolescents who were aged 14–15 years had more chronic knee pain than children who were aged 9–10 years, and more than half of the participants with chronic knee pain were involved in some form of sport. A prospective cohort study of risk factors for persistent knee pain, including 768 adolescents between 12 and 15 years old, found that a high level of sports participation was a risk factor for chronic knee pain [19]. Our study showed that female sex and high HSS Pedi-FABS were risk factors for protraction of patellar and patellar tendon pain. A study investigating differences in how children and adolescents cope with chronic pain reported that although boys and girls coped differently, there was no difference in the effects on pain [26]. We believe that the tendency of higher chronicity in girls must be investigated. We also found that younger children were at a higher risk of pain protraction than older children. This may be due to the children’s physical development stage and how these participants tend to cope with chronic pain [26].

The strength of this study is that it was a prospective cohort study, and a larger number of participants were included compared to that in previous studies. In addition, collecting knee pain data every month likely incurred less recall bias than annual studies. However, this study has some limitations. First, we evaluated knee pain based on the participants’ self-reported tenderness. Because pain is a subjective symptom, if the pain was not troubling the children, they might not have reported the presence of knee pain. In addition, we were not able to reach a definitive diagnosis. Furthermore, the duration of knee pain within each month was unclear, because the surveillance was conducted on a monthly basis. The duration of pain at recurrence may be another important factor for the definition of protraction. However, once a month was the limit for surveillance frequency, considering the burden of teachers and participants. Second, the proportion of missing knee pain data was relatively high, with 29.0% of participants lacking data on knee pain at least once during the follow-up period. This rate was calculated by omitting participants with more than one instance of missing data, which may be a strict cut-off for children and adolescents. The rate of missing data count to the total data count was only 7.1%. Therefore, the results may be biased in performing sensitivity analyses for the occurrence and protraction of knee pain. These results are shown in Additional files 1 and 2 and are similar to the initial analysis. Third, reliability tests for assessment methods were not performed. Because all physical evaluation tests were commonly used procedures in daily practice, we considered that reliability check was unnecessary. All examiners received three training sessions to standardize the evaluation method. Fourth, this study was conducted in a single public school; thus, generalizability of the results is questionable. Finally, we do not know if any of the participants had a history of temporal knee pain before participating in the study.

## Conclusions

We found that the patellar and patellar tendon pain occurrence rate per year was 22.2% among children and adolescents, with one-third of the participants developing chronic knee pain. We also showed that children and adolescents with high levels of physical activity had a higher risk for the occurrence and protraction of patellar and patellar tendon pain. In addition, girls were at higher risk of protraction than boys.

## Supplementary Information

**Additional file 1.** The odds ratio (OR) and confidence interval (CI) of occurrence of knee pain within a year by each factor in a sensitivity analysis.

**Additional file 2.** The odds ratio (OR) and confidence interval (CI) of the protraction of knee pain by each factor in a sensitivity analysis.

## Data Availability

Requests for data not shown in the body of this manuscript can be made to the corresponding author.

## Abbreviations

HBD: Heel buttock distance
SLRA: Straight leg raising angle
DFA: Dorsiflexion angle of the ankle joint with knee extension
HSS Pedi-FABS: The Hospital for Special Surgery Pediatric Functional Activity Brief Scale.
SD: Standard deviations
OR: Odds Ratio
CI: Confidence interval
