# Supplementary material for: Risk factors for the occurrence and protraction of patellar and patellar tendon pain in children and adolescents: a prospective cohort study of 3 years
Source: BMC Musculoskelet Disord. 2022 Apr 26;23:389. doi: 10.1186/s12891-022-05349-y (PMC9044608; doi:10.1186/s12891-022-05349-y)
Supplement: Supplementary file 1 — Additional file 1. The odds ratio (OR) and confidence interval (CI) of occurrence of knee pain within a year by each factor in a sensitivity analysis. [file 12891_2022_5349_MOESM1_ESM.docx]

**Appendix 1**. The OR and CI of occurrence of knee pain within a year by each factor in sensitivity analysis

|  | OR [95% CI] | P value |
| --- | --- | --- |
| Age | 0.83 [0.77, 0.90] | <0.01 |
| Sex ^a^ | 0.94 [0.68, 1.30] | 0.71 |
| BMI | 1.00 [0.93, 1.07] | 0.94 |
| HBD | 1.00 [0.94, 1.06] | 0.99 |
| SLRA ^b^ | 0.86 [0.85, 1.09] | 0.52 |
| DFA ^b^ | 1.07 [0.88, 1.10] | 0.79 |
| HSS Pedi-FABS | 1.03 [1.01, 1.05] | 0.02 |

BMI, body mass index; HBD, heel-buttock distance; SLRA, straight leg raising angle; DFA, dorsiflexion angle of the ankle joint with knee extension; HSS Pedi-FABS, The Hospital for Special Surgery Pediatric Functional Activity Brief Scale; OR, odds ratio; CI, confidence interval

a: Reference group is boys.

b: Units of SLRA and DFA are in 10° and 5° increments, respectively.
