# Supplementary material for: Risk factors for the occurrence and protraction of patellar and patellar tendon pain in children and adolescents: a prospective cohort study of 3 years
Source: BMC Musculoskelet Disord. 2022 Apr 26;23:389. doi: 10.1186/s12891-022-05349-y (PMC9044608; doi:10.1186/s12891-022-05349-y)
Supplement: Supplementary file 2 — Additional file 2. The odds ratio (OR) and confidence interval (CI) of the protraction of knee pain by each factor in a sensitivity analysis. [file 12891_2022_5349_MOESM2_ESM.docx]

**Appendix 2.** The OR and CI of the protraction of knee pain by each factor in a sensitivity analysis

|  | OR [95% CI] | P value |
| --- | --- | --- |
| Age | 0.87 [0.79, 0.95] | 0.00 |
| Sex ^a^ | 1.17 [0.81, 1.70] | 0.40 |
| BMI | 1.01 [0.93, 1.09] | 0.90 |
| HBD | 1.01 [0.94, 1.08] | 0.79 |
| SLRA ^b^ | 0.89 [0.77, 1.02] | 0.10 |
| DFA ^b^ | 1.06 [0.95, 1.18] | 0.28 |
| HSS Pedi-FABS | 1.02 [1.00, 1.05] | 0.02 |

BMI, body mass index; HBD, heel-buttock distance; SLRA, straight leg raising angle; DFA, dorsiflexion angle of the ankle joint with knee extension; HSS Pedi-FABS, The Hospital for Special Surgery Pediatric Functional Activity Brief Scale; OR, odds ratio; CI, confidence interval

a: Reference group is boys.

b: Units of SLRA and DFA are in 10° and 5° increments, respectively.
